# A Stability-Indicating High Performance Liquid Chromatographic Method for the Determination of Diacerein in Capsules

**DOI:** 10.4103/0250-474X.51946

**Published:** 2009

**Authors:** Janhavi Rao, Kanchan Chauhan, K. R. Mahadik, S. S. Kadam

**Affiliations:** Department of Pharmaceutical Chemistry, Poona College of Pharmacy, Bharati Vidyapeeth University, Erandwane, Pune - 411 038, India

**Keywords:** Diacerein, Stability-indicating, HPLC-UV

## Abstract

A stability-indicating HPLC method was developed and validated for the quantitative determination of diacerein in capsule dosage forms. An isocratic separation was achieved using a perfectsil target ODS-3, 250×4.6 mm i.d., 5 µm particle size columns with a flow rate of 1 ml/min and using a UV detector to monitor the eluate at 254 nm. The mobile phase consisted of phosphate buffer:acetonitrile (40:60, v/v) with pH 4.0 adjusted with phosphoric acid. The drug was subjected to oxidation, hydrolysis, photolysis and thermal degradation. Diacerein was found to degrade in acidic, basic, and oxidative stress and also under neutral condition. Complete separation of degraded products was achieved from the parent compound. All degradation products in an overall analytical run time of approximately 10 min with the parent compound diacerein eluting at approximately 4.9 min. The method was linear over the concentration range of 1-10 µg/ml (r^2^ = 0.9996) with a limit of detection and quantitation of 0.01 and 0.05 µg/ml respectively. The method has the requisite accuracy, selectivity, sensitivity, precision and robustness to assay diacerein in capsules. Degradation products resulting from the stress studies did not interfere with the detection of diacerein and the assay is thus stability-indicating.

Diacerein 1,8-diacetoxy-3-carboxy anthraquinone[1] (fig. 1) is a novel osteoarthritis drug which selectively inhibits the IL-1. It is semisynthetic anthraquinone derivative extracted from certain plants. It directly inhibits IL-1 synthesis and release which plays a fundamental role in osteoarthritis pathophysilogy and cartilage destruction. IL-1 promotes expression of inducible nitric oxide synthase, increase release of PGE_2,_ Stimulates matrix metalloproteinase, IL-6, IL-8 in human osteoarthritis chondrocytes, which promotes joint degradation. Hence, by inhibiting IL-1 diacerein retards all pathological prepossess initiated OA[2–4]. Diacerein also inhibits IL-1 induced expression of cartilage degrading enzymes. In contrast to NSAIDS, diacerein does not inhibit synthesis of prostaglandin hence no gastrodeodenal toxicity. It also involved in prevention of loss of hydroxyproline and proteoglycans in joint cartilage[3,4]. After thorough literature survey only one HPLC method was found to be reported[5]. But the reported method was found to be more time consuming and more solvent consuming as it shows the long retention time for pure drug. The proposed validated method is more economical, precise, accurate and specific for the quantitative determination of diacerein in pharmaceutical dosage form.

**Fig. 1:**
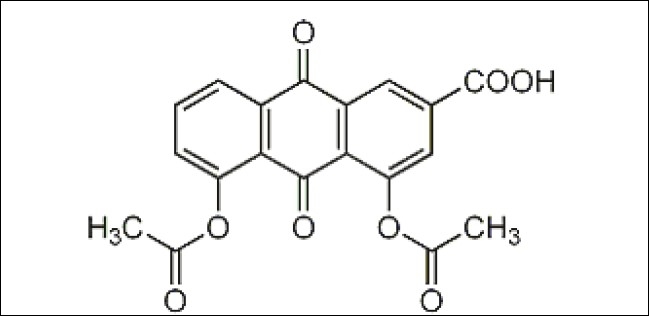
Structural formula of diacerein.

## MATERIALS AND METHODS

Diacerein pure compound was kindly supplied by Glenmark Pharmaceuticals, Kurkumbh, India and was used without further purification. Diacerein capsules, (Artodar) containing 50 mg diacerein as per label claim were purchased from local pharmacy. All the chemicals used were of analytical grade. Purified HPLC grade water was obtained by double distillation and filtered through filter (Millipore, Milford, MA) and was used to prepare all the solutions.

### HPLC instrumentation and conditions:

The HPLC system consists of a pump (Jasco PU-2080, intelligent HPLC pump) with injecting facility programmed at 20 µl capacity per injection was used. The detector consists of a UV/Vis (Jasco UV 2075) operated at a wavelength of 254 nm. The software used was Jasco Borwin version 1.5, LC-Net II/ADC system. The chromatographic separation was performed using a Perfectsil® ODS-3, 5µm, 250×4.6 mm i.d. column. Separation was achieved using a mobile phase consisting of phosphate buffer-acetonitrile (40:60 v/v) solution at a flow rate of 1 ml/min. The eluent was monitored using UV detection at a wavelength of 254 nm. The column was maintained at ambient temperature and injection volume of 20 µl was used. The mobile phase was filtered through 0.45 µm micron filter prior to use.

### Preparation of stock and standard solutions:

A stock solution of diacerein (1 mg/ml) was prepared by accurately weighing approximately 10 mg of diacerein into 10 ml volumetric flask and dissolved in 5 ml of DMSO and was made up to the volume with HPLC grade acetonitrile. The stock solution is protected from light using aluminium foil and stored for one week and was found to be stable during this period. Aliquots of the standard stock solutions of diacerein were transferred using A-grade bulb pipettes into 10 ml volumetric flasks and solutions were made up to the volume with mobile phase to give the final concentrations of 1-10 µg/ml.

### Estimation of diacerein from pharmaceutical dosage form:

To determine the content of diacerein in capsules (label claim: 50 mg diacerein) 20 capsules were opened and the contents were weighed and mixed. An aliquot of powder equivalent to the weight of 1 capsule was accurately weighed and transferred to 100 ml volumetric flask and was dissolved in 5 ml of DMSO and made up to the volume with HPLC grade acetonitrile. The volumetric flask was sonicated for 30 min to affect complete dissolution. The solutions were filtered through a 0.45 µm nylon filter. Suitable aliquots of the filtered solution was added to a volumetric flask and made up to the volume with mobile phase to yield the concentrations of 3, 5 and 7 µg/ml. A 20 µl volume of each sample solution was injected into HPLC, six times, under the conditions described above. The peak areas were measured at 254 nm and concentrations in the samples were determined by comparing the area of sample with that of the standard.

### Forced degradation studies[6]:

In order to determine whether the analytical method and assay were stability-indicating, Diacerein pure drug was stressed under various conditions to conduct forced degradation studies. As diacerein was insoluble in water, chloroform and methanol, DMSO was used to dissolve the drug and then volume was made up with acetonitrile. A stock solution of 100 µg/ml was prepared by dissolving 10 mg of diacerein in 5 ml of DMSO and volume was made up to 100 ml with acetonitrile. This solution was used for forced degradation studies to evalute the stability indicating property and specificity of proposed method. In all degradation studies the average peak area of standard diacerein and degradation sample after application (20 μg/ml for HPLC) of six replicates were obtained.

### Oxidation:

To 2 ml of stock solution, 2 ml of 1% hydrogen peroxide was added separately. The solutions were kept for 30 min at room temperature. For HPLC study, the resultant solution was diluted to obtain 20 μg/ml solution and 20 μl were injected into the system and the chromatograms were recorded to assess the stability of sample.

### Acid Degradation Studies:

To 2 ml of stock solution, 2 ml of 0.01 N hydrochloric acid was added. The solution was kept for 15 min at room temperature. The resultant solution was diluted to obtain 20 μg/ml solution and 20 μl were injected into the system and the chromatograms were recorded to assess the stability of sample.

### Alkali Degradation Studies:

To 2 ml of stock solution, 2 ml of 0.01 N sodium hydroxide was added. The solution was kept for 15 min at room temperature. The resultant solution was diluted to obtain 20 μg/ml solution and 20 μl were injected into the system and the chromatograms were recorded to assess the stability of sample.

### Neutral Degradation Studies:

Stress testing under neutral conditions was studied by refluxing the drug in water for 3 h at a temperature of 70°. For HPLC study, the resultant solution was diluted to 20 µg/ml solution and 20 µl were injected into the system and the chromatograms were recorded to assess the stability of the sample.

### Dry Heat Degradation Studies:

The standard drug was placed in oven at 80° for 6 h to study dry heat degradation. For HPLC study, the resultant solution was diluted to 20 µg/ml solution and 20 µl were injected into the system and the chromatograms were recorded to assess the stability of the sample.

### Photo Stability studies:

The photochemical stability of the drug was also studied by exposing the stock solution (1 mg/ml) to direct sunlight for 360 h. on a wooden plank and kept on terrace. For HPLC study, the resultant solution was diluted to obtain 20 μg/ml solutions and 20 μl were injected into the system and the chromatograms were recorded to assess the stability of sample.

## RESULTS AND DISCUSSIONS

The HPLC procedure was optimized with a view to develop a stability indicating assay method. Pure drug along with its degraded products were injected and run in different solvent systems. Initially methanol and water and acetonitrile and water in different ratios were tried. It was found that acetonitrile and water system gives good results than methanol and water as the drug was more soluble in acetonitrile than methanol. Acetonitrile:water in the ratio of 80:20 was not able to give good peak symmetry with acceptable retention time. An attempt to improve peak symmetry was made by adding phosphate buffer to the mobile phase. The presence of phosphate buffer in mobile phase resulted in excellent overall chromatography with appropriate peak symmetry and complete base line resolution. Finally the mobile phase consisting of phosphate buffer and acetonitrile (40:60 v/v) with pH 4.0 adjusted with phosphoric acid was selected for validation purpose and stability studies. The method was validated with respect to parameters including linearity, limit detection (LOD), limit of quantitation (LOQ), recovery, precision, accuracy, robustness and selectivity and a summary of validation parameters were presented in Table 1.

**TABLE 1:** SUMMARY OF VALIDATION PARAMETERS

Parameters*	HPLC
Linearity range	1-10
Correlation coefficient	0.9996
Limit of detection	0.01
Limit of quantitation	0.05
Recovery	99.23
Repeatability	1.28
Intermediate precision	1.30

* Average of six determinations.

Diacerein showed linearity in the concentration range of 1-10 µg/ml (r^2^ = 0.9996±0.25) for HPLC. Linearity was evaluated by determining ten standard working solutions containing 1-10 µg/ml thrice in triplicate. Peak areas of diacerein were plotted versus diacerein concentration and linear regression analysis performed on the resultant curve. For HPLC method the linearity of calibration graphs and adherence of the system to Beer's law was validated by high value of correlation coefficient and the standard deviation for intercept value was less than 2%.

The LOD and LOQ were determined based on a signal-to-noise ratios and were based on analytical responses of 3 and 10 times the background noise, respectively. The LOD was found to be 0.01 μg/ml. The LOQ was found to be 0.05 μg/ml.

Proposed method when used for extraction and subsequent estimation of diacerein from pharmaceutical dosage form after spiking with additional drug, afforded recovery of 98.21 to 100.05% and mean recovery of diacerein from the marketed formulation are listed in Table 2.

**TABLE 2:** RECOVERY OF DIACEREIN

Level (%)	Actual concentration added (μg)	Observed concentration (μg)	% Recovery±SD*
0	5	4.96	99.21±0.13
80	4	3.93	98.29±0.26
100	5	4.97	99.45±0.08
120	6	6.003	100.05±0.06

* SD: standard deviation.

The precision of assay was determined with respect to both repeatability and reproducibility. An amount of the product powder equivalent to 100% of the label claim of diacerein was accurately weighed and assayed. System repeatability was determined by six replicate applications and six times measurement of a sample solution at the analytical concentration. The repeatability of sample application and measurement of peak area for active compound were expressed in terms of % RSD (relative standard deviation). Method repeatability was obtained from RSD value by repeating the assay three times in same day for intra-day precision. Inter-day precision was assessed by the assay of three sample sets on different days (inter-day precision). The intra-day and inter-day variation for determination of diacerein was carried out at three different concentration levels 3, 5, 7 µg/ml (Table 3).

**TABLE 3:** INTRA-DAY AND INTER-DAY PRECISION

Concentration	Intra-day (μg/ml)	%RSD*	Inter-day (μg/ml)	%RSD* (μg/ml)
3	2.99	0.52	2.95	1.22
5	4.92	1.36	4.95	1.08
7	6.87	1.42	6.91	1.62

* %RSD: percent relative standard deviation.

The accuracy of assay was determined by interpolation of replicates (n=6) peak areas of three accuracy standards (3, 5, 7 μg/ml) from a calibration curve prepared as previously described. In each case the accuracy was calculated. The resultant concentrations were 2.94±0.06, 4.93±0.07 and 7.06±0.06 (mean±standard deviation), respectively.

To evaluate HPLC method robustness a few parameters were deliberately varied. The parameters included variation of C_18_ columns from different manufacturers, flow rate, percentage of acetonitrile in the mobile phase, column temperature and acetonitrile of different lots. Each parameters (except columns from different manufacturers and solvents of different lots) were changed at three levels (-1, 0 and 1) and examined. One factor at the time was changed to estimate the effect. Thus, replicate injections (n=6) of standard solution at three concentration levels were performed under small changes of six chromatographic parameters (factors). Results indicate that the selected factors remained unaffected by small variations of these parameters. The results from the two columns indicated that there is no significant difference. It was also found that acetonitrile of different lots from the same manufacturer had no significant influence on the determination. Insignificant differences in peak areas and less variability in retention time were observed.

**Fig. 2:**
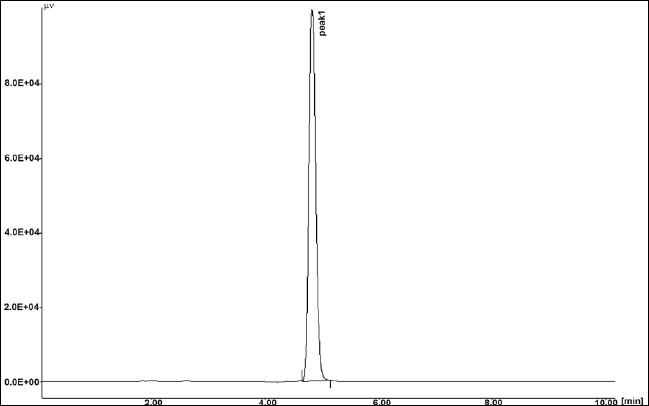
Chromatogram of standard drug Mobile phase phosphate buffer:acetonitrile (40:60 v/v). Peak 1 standard drug (20 μg/ml); (t_R_: 4.9±0.028), 254 nm.

**Fig. 3:**
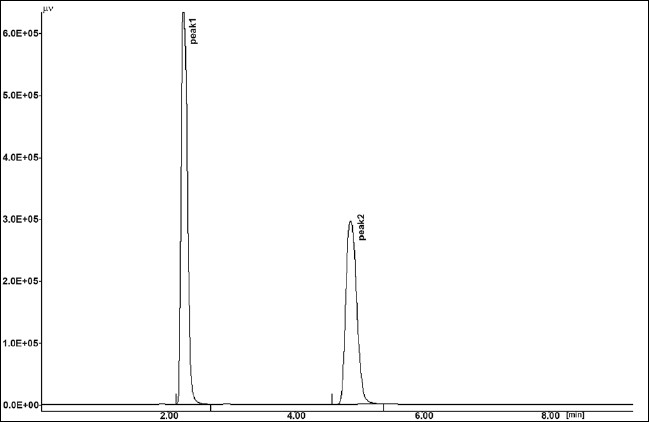
Chromatogram of hydrogen peroxide. 1.0 %w/v, kept for 30.0 min at room temperature, treated diacerein; peak 1 degraded (t_R_:2.34 min), peak 2 standard (t_R_:4.91 min).

**Fig. 4:**
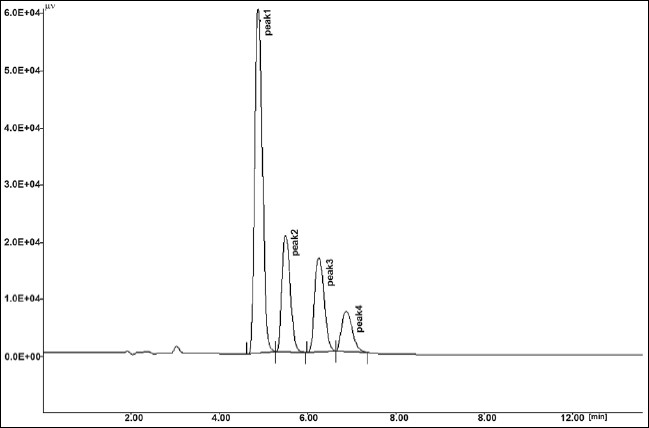
Chromatogram of acid hydrolysis. Kept for 15 min at room temperature, treated diacerein; Peak 1 standard (t_R_: 4.91 min), Peak 2 degraded (t_R_: 5.72 min), Peak 3 degraded (t_R_: 6.2 min), Peak 4 degraded (t_R_: 6.9 min).

**Fig. 5:**
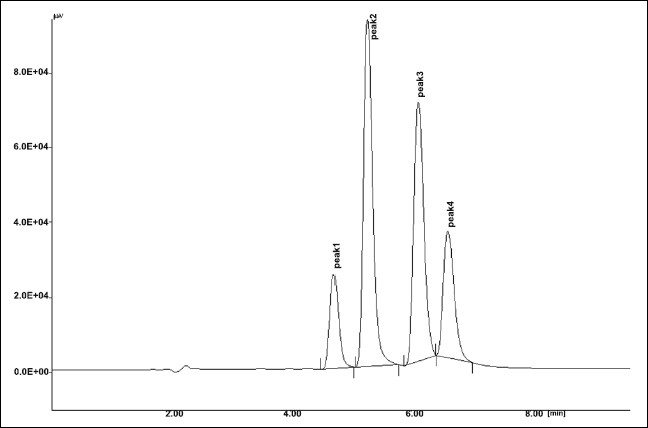
Chromatogram of base hydrolysis. Kept for 15 min at room temperature, treated diacerein; Peak 1 standard (t_R_: 4.8 min) Peak 2 degraded (t_R_: 5.62min), Peak 3 degraded (t_R_: 6.1 min), Peak 4 degraded (t_R_: 6.8 min).

**Fig. 6:**
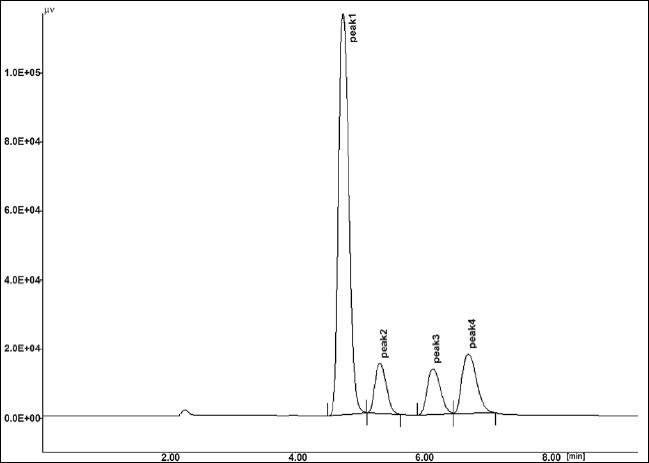
Chromatogram of neutral hydrolysis. Kept for 15 min at room temperature, treated diacerein; Peak 1 standard (t_R_: 4.8 min), Peak 2 degraded (t_R_: 5.3 min), Peak 3 degraded (t_R_: 6.2 min), Peak 4 degraded (t_R_: 6.8 min).

The results of stress testing studies indicated a high degree of selectivity of this method for diacerein. Typical chromatograms obtained from the assay of pure sample and stressed samples are shown in figs. 2, 3, 4, 5 and 6, respectively. The average retention time±standard deviation for diacerein was found to be 4.9±0.018 for six replicates. The peaks obtained were sharp and have clear baseline separation.

Diacerein is characterized by an anthraquinone moiety with a side chain of acetoxy group and carboxylic acid group, which was prone to hydrolysis. All the main degradation products were separated from the parent compound[6,7]. Diacerein was found to be stable under dry heat conditions and also no decomposition was seen on exposure of solid drug powder to light, which was kept in day light for 360 h. The drug was unstable under basic stress conditions when kept for 15 min under room temperature. The drug was degraded approximately to 90%. Also it was unstable in acidic conditions when kept for 15 min at room temperature. The drug was degraded approximately to 48%. When kept under oxidative stress conditions with 1% H_2_ O_2_ for 30 min at room temperature, the drug was degraded to around 54%. In neutral conditions when the drug was refluxed with water for 3 h, around 30% degradation was shown. The stability of stock solution was determined by quantitation of diacerein and comparison to freshly prepared standard. No significant change was observed in the stock solution response, relatively to freshly prepared standard (Table 4).

**TABLE 4:** STRESSED STUDY DATA OF DIACEREIN

Condition	Time (h)	% Degradation	*1t_R(min)_ of degradation products
H_2_O_2_ 1% (RT)*2	30 min	54%	2.34
0.01N HCL (RT)	15 min	48%	5.72, 6.2, 6.9
0.01N NaOH (RT)	15 min	90%	5.62, 6.1, 6.8
Neutral hydrolysis	3 h	30%	5.3, 6.2, 6.8
reflux (70°)			
Dry heat (80°)	6 h	----	----
Day light (25°)	360 h	----	----
Wet heat, reflux (70°)	6 h	----	----

*1 retention time in minutes.

*2 room temperature.

The proposed method was applied to the determination of diacerein in Artodar capsules. The result of these assay yielded 99.68±0.62% (%RSD=0.86) of label claim of the capsules. The results of the assay indicate that the method is selective for the assay of diacerein without interference from the excipients used in these capsules (Table 5).

**TABLE 5:** DETERMINATION OF DIACEREIN IN CAPSULE DOSAGE FORM

Parameter	Value
Label claim mg/capsule	50 mg
Drug content %± SD*1	99.68±0.62
%RSD*2	0.86

*1 standard deviation.

*2 relative standard deviation.
